# Multi-Wire Tri-Gate Silicon Nanowires Reaching Milli-pH Unit Resolution in One Micron Square Footprint

**DOI:** 10.3390/bios6010009

**Published:** 2016-03-15

**Authors:** Enrico Accastelli, Paolo Scarbolo, Thomas Ernst, Pierpaolo Palestri, Luca Selmi, Carlotta Guiducci

**Affiliations:** 1 Institute of Bioengineering, École Polytechnique Fédérale de Lausanne (EPFL), 1015 Lausanne, Switzerland; enrico.accastelli@epfl.ch; 2 DIEGM, Università degli Studi di Udine, 33100 Udine, Italy; scarbolo.paolo@spes.uniud.it (P.S.); palestri@uniud.it (P.P.); luca.selmi@uniud.it (L.S.); 3 Laboratoire d’Électronique et de Technologie de l’Information (LETI), Commissariat à l’Énergie Atomique et aux Énergies Alternatives (CEA), 38054 Grenoble Cedex 9, France; thomas.ernst@cea.fr

**Keywords:** silicon nanowires, tri-gate transistors, ISFET, pH sensors, 1/f noise

## Abstract

The signal-to-noise ratio of planar ISFET pH sensors deteriorates when reducing the area occupied by the device, thus hampering the scalability of on-chip analytical systems which detect the DNA polymerase through pH measurements. Top-down nano-sized tri-gate transistors, such as silicon nanowires, are designed for high performance solid-state circuits thanks to their superior properties of voltage-to-current transduction, which can be advantageously exploited for pH sensing. A systematic study is carried out on rectangular-shaped nanowires developed in a complementary metal-oxide-semiconductor (CMOS)-compatible technology, showing that reducing the width of the devices below a few hundreds of nanometers leads to higher charge sensitivity. Moreover, devices composed of several wires in parallel further increase the exposed surface per unit footprint area, thus maximizing the signal-to-noise ratio. This technology allows a sub milli-pH unit resolution with a sensor footprint of about 1 µm^2^, exceeding the performance of previously reported studies on silicon nanowires by two orders of magnitude.

## 1. Introduction

Reducing the size of electronic devices affects the signal-to-noise ratio (SNR). Structures with improved gate-to-channel coupling, indicated as multi-gate devices, were conceived for traditional solid-state systems to overcome this issue [1]. In particular, the feature of these devices, enhanced subthreshold swing, reduced short-channel effects such as drain-induced barrier lowering (DIBL) and, consequently, resulted in an improved scalability [2,3,4]. As a matter of fact, the introduction of multi-gate device architectures has so far effectively sustained Moore’s law and kept up the pace of technological advancement in the electronics industry.

In the field of bioanalytics, the large-scale integration of CMOS chips exposing arrays of liquid-gate FET pH-sensors has shown its potential in applications such as DNA sequencing [5], and holds the promise to innovate DNA quantification based on quantitative PCR [6,7,8]. If the paradigm of nanoscale multi-gate devices improved as well the SNR of liquid-gate sensors, it would be possible to reduce the sensor size and consequently increase the throughput of analytical systems.

Silicon-based multi-gate structures, commonly indicated as nanoribbons (SiNRs) and nanowires (SiNWs), have been proposed as bio and chemical sensors [9,10,11,12,13,14,15,16,17,18,19,20,21,22,23,24,25,26,27,28,29] . Although a few works indicate that improved macromolecular sensing can be achieved with smaller devices [30], no consensus has been reached on the effect of size reduction on pH sensitivity as well as the role of device technology, device layout, bias and signal readout [31,32]. In what follows, we show that tri-gate silicon nanoribbons with rectangular cross-section exhibit an enhanced sensitivity *vs*. pH when decreasing their width below a few hundreds of nanometers. We perform noise characterization for devices of different size and biasing, proposing a multi-wire configuration to achieve higher SNR. The resolution that can be achieved with a sensor footprint of one micrometer square corresponds to a 0.8‰ shift in pH, which is equivalent to approximately 100 electrons in the case of silicon dioxide interfaces. This performance is two orders of magnitude better than the one obtained with planar devices [33,34].

We developed tri-gate devices on Silicon-On-Insulator (SOI) substrates based on self-aligned CMOS-compatible top-down processes, with accuracy down to 5 nm. The use of a semi-industrial process ensures a high level of control over process variations and enables the fabrication of high-density individually connected SiNRs with a well-defined orientation and a wide range of widths and lengths. Such features still represent a challenge for bottom-up technologies [35].

## 2. Experimental Section

### 2.1. Technology and Fabrication Process

**Figure 1:**
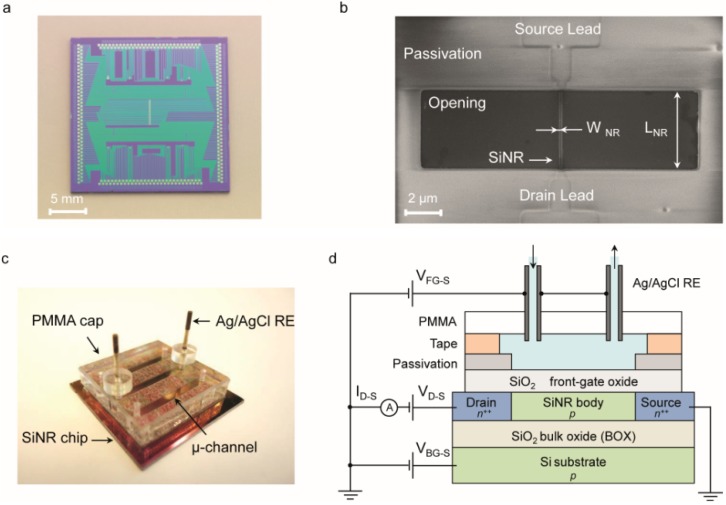
(**a**) Optical image of the chip; (**b**) SEM image of a Silicon NanoRibbon (SiNR). Top view of a device with *W*_NR_ = 87 nm and *L*_NR_ = 4250 nm. The source and drain leads are passivated, while the body of the device is exposed. The length of each SiNR corresponds to the opening in the passivation; (**c**) Optical image of the microfluidics setup; (**d**) Cross section of the fabricated device and a sketch of the measurement setup (not to scale).

The n+/p/n+ SiNRs are fabricated on SOI wafers and patterned by means of a top-down hybrid deep ultraviolet (DUV) and e-beam lithography followed by a reactive ion etching (RIE). The fabrication process was entirely developed at CEA-LETI. The wafers are composed of a 200 nm silicon layer on top of a 400 nm thick SiO_2_ insulating bulk oxide layer (BOX). The silicon top layer is first thinned by chemical mechanical polishing (CMP) and then by several steps of silicon thermal oxidation/desoxidation to obtain a 50 nm silicon top layer, which defines the thickness (tNR) of the SiNRs. Devices with feature sizes above 300 nm are patterned using deep ultraviolet (DUV), while e-beam lithography is employed for smaller dimensions. Once patterned, the undesired material is etched away by reactive ion etching (RIE). A high quality 3 nm SiO_2_ gate oxide is grown using thermal dry oxidation. In order to obtain n-type devices, the SiNRs are doped by boron implantation (1 × 10^18^ cm^−3^). A sacrificial polysilicon gate is used as a hard mask to dope the source and drain terminals with phosphorous (1 × 10^19^ cm^−3^), thus effectively defining the SiNR length. The entire chip surface, with the exception of the contact pads and the SiNR, is passivated by a multi-layer insulator composed of 50 nm of Si_3_N_4_, 300 nm of tetraethyl orthosilicate (TEOS) and 200 nm of phosphosilicate glass (PSG) (Figure 1b). This multilayer insulator is designed to withstand the liquid environment and to avoid the propagation of pin-holes in the passivation that could short-circuit the electrolyte with the contact lines [36].

### 2.2. Ag/AgCl Reference Electrode Fabrication

The Ag/AgCl electrodes are obtained by a galvanostatic oxidation on silver tubes. The silver tubes are dipped in 0.1 M HNO_3_ for about 1 min, in order to clean and activate the surface, and then rinsed with deionized water. After the surface activation, the silver wire is connected to a working electrode (anode); a platinum wire is connected to a counter electrode (cathode) and both of them are dipped into a solution of 0.1 M KCl. A current density of 2.5 mA/cm^2^ is applied at the anode for a time that depends on the desired thickness of the AgCl layer (5 µm in this work). At the end of the oxidation, the Ag/AgCl tubes are dipped into a solution of 0.1 M KCl for at least 4 hours prior to use. The performances in terms of voltage drift have been compared with respect to commercial Ag/AgCl (see Supplementary Figure S10).

### 2.3. Microfluidics

The microchannels are obtained with a chemical-resistant double-coated tape (3M 9086), patterned by laser micromachining. The height of the channels is thereby defined by the thickness of the tape (190 μm). A poly(methyl methacrylate) (PMMA) cap with holes drilled in the locations of inlets and outlets is placed on top of the tape to seal the channels, and the inlet and outlet tubes are inserted.

A syringe pump (Harvard Apparatus) is used to flux the solution of interest into the microfluidics. Due to the double-coated tape system, the microfluidics can easily be removed by incubating the chip in 2-Propanol alcohol overnight. The chip can, therefore, be cleaned more thoroughly and re-used. Figure 1b shows an SEM image of a used device after the cleaning procedure.

### 2.4. Electrical Characterization

The electrical characterization of the devices was performed by means of a semiconductor parameter analyzer (SPA, Agilent 4156C) used both in sweep and sampling modes, controlled by LabVIEW VIs and MATLAB scripts via a GPIB interface.

### 2.5. pH Sensing Experiments

The SiNR chips are cleaned by successive washing steps of ethanol/acetone/ethanol (10 min each), separated by rinsing with deionized water and drying with N_2_. After the cleaning procedure of the chip surface, the microfluidic module is mounted and the chip is ready for the experiment. The solutions at different pH are interspersed by 10 µM KCl (pH_KCl_ ~6.8) injections. Each injection is 15 min long, at fixed flow rate of 5 µL/min. At the end of each injection, the syringe pump is stopped and the solution is changed.

The different pH solutions are prepared starting with 10 mM phosphate buffered saline (PBS) containing 100 mM KCl, which is added to stabilize the Ag/AgCl RE. The solutions are adjusted to the desired pH by adding either a strong acid (H_2_SO_4_) or a strong base (NaOH), so that the difference in the ionic strength of the solutions results to be negligible.

## 3. Results and Discussion

### 3.1. pH Sensitivity of Single SiNR Devices as a Function of Their Width

The fabricated chip features 75 SiNRs with thickness (*t*_NR_) of 50 nm and different widths (*W*_NR_) and lengths (*L*_NR_) (Figure 1a,b). Devices with identical *L*_NR_ and different *W*_NR_ (and vice versa) were fabricated with the specific aim of studying the effect of size on the device noise and sensitivity, both in terms of Δ*V*_TH_/ΔpH and Δ*I*_D-S_/ΔpH.

The SiNR chip is coupled with a microfluidic module that employs Ag/AgCl reference electrodes (REs) and allows subsequent injections of different solutions into sub-5 µL microchannels (Figure 1c) [37].

The SiNRs can be operated either by the solid-state back-gate or by the liquid front-gate (Figure 1d). The back-gating approach consists in applying a gate voltage (*V*_BG-S_) between the silicon substrate and the source terminal. Instead, in the case of front-gating, a voltage *V*_FG-S_ is applied between the electrolyte solution, biased by Ag/AgCl REs, and the source terminal, while the back-gate is short-circuited with the source contact. As shown in our previous work [37], the front-gating configuration with grounded back-gate prevents from any parasitic coupling between the electrolyte solution and the environment.

**Figure 2:**
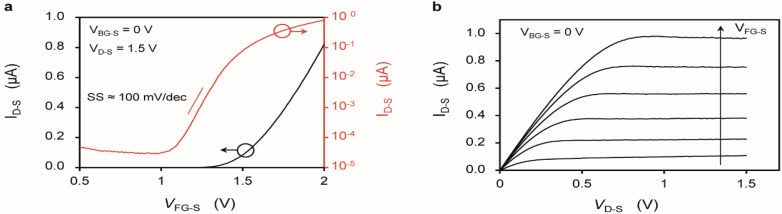
(**a**,**b**) Characterization of a SiNR with length (*L*_NR_) of 2375 nm and a width (*W*_NR_) of 290 nm. The electrolyte is 100 µM KCl. (**a**) Drain current (*I*_D-S_) *vs.* gate voltage (*V*_FG-S_) plotted in linear (left *y* axis) and logarithmic (right *y* axis) scale; (**b**) Drain current (*I*_D-S_) *vs.* drain voltage (*V*_D-S_) plotted for different constant gate voltages (*V*_FG-S_), ranging from 1.5 V to 2.0 V, with a step of 0.1 V.

**Figure 3:**
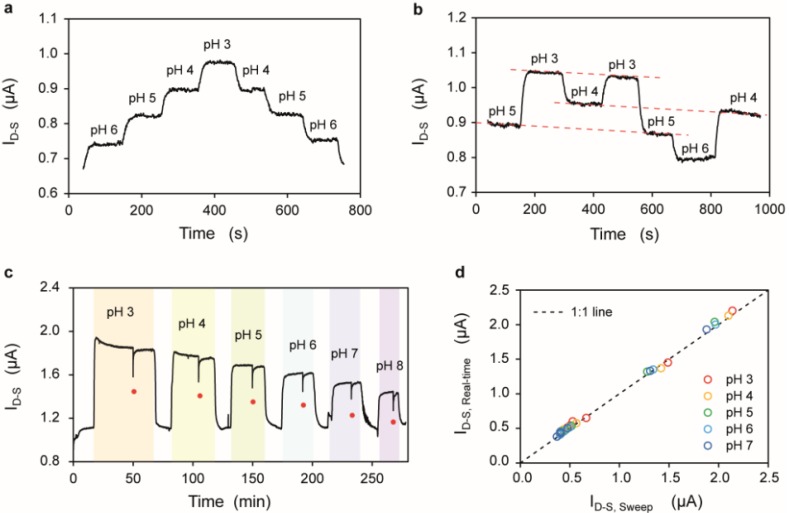
(**a**) Drain current (*I*_D-S_) observed at fixed voltage bias upon injection of decreasing/increasing series of pH values; (**b**) Drain current (*I*_D-S_) observed at fixed voltage bias upon injection of randomize series of pH values. The three parallel red dotted lines highlight the drift affecting the *I*_D-S_; (**c**) Drain current (*I*_D-S_) measurement upon injection of solutions with different pH values and identical ionic strength. The red dots indicate when the *I*_D-S_-*V*_FG-S_ characterization is performed; (**d**) Average *I*_D-S_ observed at fixed polarization (*I*_D-S,Real-time_) *vs.* the *I*_D-S_ extracted from the *I*_D-S_-*V*_FG-S_ sweeps (*I*_D-S, Sweep_) from a set of SiNRs. A 1:1 line is plotted as reference. All measurements of the figure refer to the same device which has a *W*_NR_ of 50 nm and a *L*_NR_ of 2375 nm.

Figure 2 shows the *I*_D-S_-*V*_FG-S_ and *I*_D-S_-*V*_D-S_ characteristics of a SiNR in front-gating bias. The SiNR shows a MOSFET behavior, with threshold voltage (*V*_TH_) of approximately 1.5 V and a sub-threshold swing (SS) of about 100 mV/dec. The *V*_TH_ does not show any systematic trend *vs.* W. Typical *I*_D-S,ON_/I_D-S,OFF_ ratio in virgin devices is of about 10^4^, and can be as good as 10^6^, while the leakage in Figure 2a (red curve) is more representative of a device that has been extensively used in liquid environment. Note that the leakage may have different sources, both inside and outside the active device area, but in any case does not affect our sensitivity data that is taken well above *V*_TH_. A slightly negative differential output resistance is sometimes observed at large *V*_GS_ (Figure 2b) probably due to a small drift *vs.* time (also visible in Figure 3b and discussed later on), that lowers *I*_D-S_ while sweeping the *V*_D-S_ with an integration time of 20 ms and 151 bias points per *I*_D-S_-*V*_D*-*S_ curve. A *V*_DS_ of 1.5V was selected to maximize the absolute change of the drain current with the pH without incurring in reliability issues (see also Supplementary Figure S1) and because it is favorable in terms of SNR with respect to lower *V*_DS_ values [38].

In our setup the liquid-gate terminal is common to all the devices while the source and drain contacts can be individually addressed for each SiNR device, allowing the current of the single devices to be measured independently.

Figure 3a shows the drain current of a SiNR measured in real-time upon the injection of solutions with decreasing and then increasing values of pH. According to the site binding theory [39,40,41], solutions with different pH values in contact with the gate oxide induce changes in the surface charge of the front-gate oxide as an effect of the deprotonation (protonation) of the silanol groups in the presence of OH^−^ (H^+^) ions in the electrolyte. In particular, the surface of the front-gate oxide becomes negatively charged when it is in contact with electrolyte solutions with pH values higher than its isoelectric point (pI ≈ 2 in the case of SiO_2_). The change in surface charge affects the surface potential, which in turn modifies the conductance of the device when subjected to a constant voltage bias. The device is biased above threshold (*V*_FG‑S_ − *V*_TH_ ≈ 0.5 V) and in saturation regime (*V*_D-S_ = 1.5 V), thus increasing the absolute value and the linearity of the current response in terms of Δ*I*_D-S_/ΔpH with respect to the subthreshold regime (see Supplementary Figure S1). Moreover, the devices are biased to work in the saturation regime to maximize the dynamic range. As expected in the case of an *n*-type device, the drain current decreases with increasing pH, as more and more negative charges accumulate at the oxide surface, thus depleting the electrons in the channel. The device shows little hysteresis between the forward and backward pH sweeps (see Supplementary Figure S2). Non-monotonically increasing/decreasing pH series (Figure 3b) show that the conductance of the device depends on pH and experiences a linear drift, which does not show any relevant correlation with the pH values.

Figure 3c,d compares the device current evaluated either by keeping the device at a fixed voltage bias, or by performing a sequence of *I*_D-S_-*V*_FG-S_ characteristics. Two conditioning injections at acidic and basic pH (pH 3 and pH 8, respectively) are performed at the beginning of the experiment in order to prime the front-gate oxide surface, and with the effect of improving the subthreshold slope (typically from between 150 and 200 mV/dec down to 100 mV/dec) and setting it to a stable value. For the rest of the entire experiment, the subthreshold slope remains constant, leading to a rigid shift of the *I*_D-S_-*V*_FG-S_ characteristics upon pH injections (see Supplementary Figure S3). In order to cope with the dependence of the device current with the ionic force of the electrolyte (see Supplementary Figure S4), the pH solutions were prepared to have the same ionic strength (100 mM). The monitoring of the drift was performed by exposing the device to subsequent injections of a saline solution (10 µM KCl) alternated with the solutions at different pH.

In this experiment, the device has been kept at the above mentioned bias (*V*_FG-S_ − *V*_TH_ ≈ 0.5 V, *V*_D-S_ = 1.5 V) (current *vs.* time plotted in Figure 3c), with the exception of the time points indicated by the red dots on the plot, when the device has been subjected to a series of three *V*_FG-S_ sweeps (from 0 V to 2 V). The plot on Figure 3d shows a 1:1 correlation between the current measured at a fixed bias *I*_D-S,Real-time_ (averaged over 5 min) and the current extracted from the *I*_D-S_-*V*_FG-S_ corresponding to the same bias (*I*_D-S, Sweep_).

In our measurements the current drift is not affected by the size of the devices; rather, it can be ascribed to the wear out of the Ag/AgCl reference electrode, and possibly to the relatively thin nanowire top-oxide (3 nm).

**Figure 4:**
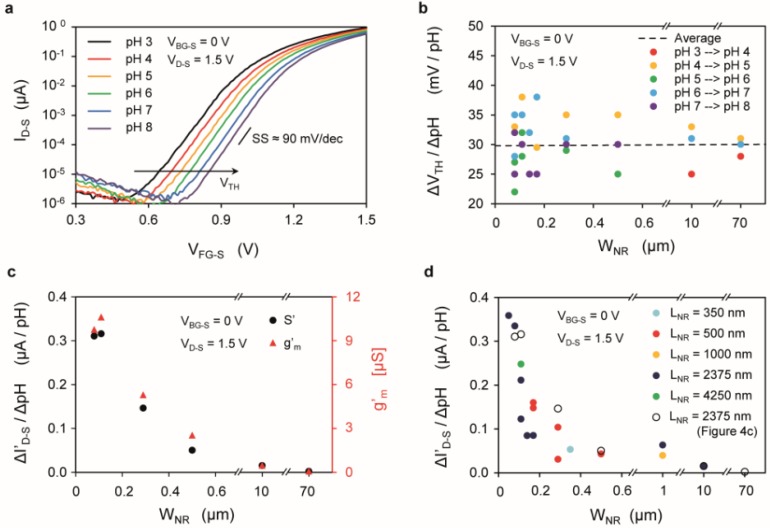
(**a**) Drain current (*I*_D-S_) *vs.* front-gate voltage (*V*_FG-S_) characteristics for a device with *W*_NR_ = 170 nm and *L*_NR_ = 2375 nm exposed to different pH; (**b**) Threshold voltage shifts over different pH, calculated at *I*_D-S_ = 1 nA and plotted against *W*_NR_; (**c**) pH sensitivity (Δ*I*’_D-S_/ΔpH) and normalized transconductance (*g*’_m_), plotted against the SiNR width (*W*_NR_). The length *L*_NR_ of the devices is constant (*L*_NR_ = 2375 nm); (**d**) Normalized-current sensitivity (S’ = Δ*I*’_D-S_/ΔpH) plotted against the SiNR width (*W*_NR_), for devices with different length (*L*_NR_), located on the same chip, all extracted at the same *V*_FG-S_ − *V*_TH_ = 0.5 V.

The experiment described in Figure 3c has been performed on several nanowires to assess their pH sensitivity, both in terms of the change of the threshold voltage Δ*V*_TH_/ΔpH (Figure 4a,b) and in terms of the normalized current signal Δ*I’*_D-S_/ΔpH (Figure 4c,d), where *I*’_D-S_ = *I*_D-S_/[(*W*_NR_ + 2·*t*_NR_)/*L*_NR_] is the current normalized by the form factor. Figure 4a shows the shift of *I*_D-S_-*V*_FG-S_ characteristics for one of the devices (*W*_NR_ = 170 nm and *L*_NR_ = 2375 nm). The threshold voltage of the *n*-type device increases due to the accumulated negative charges at the interface between the gate oxide and the electrolyte. The threshold voltage sensitivity Δ*V*_TH_/ΔpH for a set of SiNRs with different widths and identical length (*L*_NR_ = 2375 nm) located close to each other on the chip area is shown in Figure 4b. The average voltage shift is about 30 mV/pH, in agreement with other previous works using SiO_2_ as gate oxide [42,43,44]. The voltage shift does not depend on the device width (Figure 4b), as shown previously [31], while it has a well-proven dependence on the buffer capacity of the gate insulator [39].

The sensitivity in terms of current signal as a function of the device width is shown in Figure 4c,d. In this analysis the effect of the source (*R*_S_) and drain (*R*_D_) parasitic series resistances on the performance of SiNRs devices has been taken into account by extracting their value and compensating for the corresponding voltage losses (see Supplementary Figure S5). The sensitivity of Δ*I’*_D-S_/ΔpH is not constant, but instead increases for decreasing width (*W*_NR_), due to the larger device transconductance *g*’_m_ (Figure 4c). The sensitivity for devices with different widths and different lengths is plotted in Figure 4d as a function of the width, indicating that the length does not affect the sensitivity for the considered range (350–4250 nm). SiNRs with width below few hundreds of nanometers exhibit a higher transconductance.

Interestingly, this result is in contrast with other works [32], in which a linear dependence of the transconductancewith the factor (*W*_NR_ + 2·*t*_NR_)/*L*_NR_ is found. The enhancement of the transconductance with smaller *W*_NR_ could be a signature of marked corner effects, typical of devices with high body doping and rectangular cross-section [45], as opposed to low-doped trapezoidal wires [32].

The next section analyzes the noise characteristics of the SiNRs, which is essential to evaluate the dependence of the sensor performance on their size and, ultimately, to determine the smallest pH change detectable per unit footprint surface.

### 3.2. Noise Characterization of the SiNRs

At low frequencies, the noise of SiNWs and SiNRs is dominated by flicker noise [22,25,46,47], which is characterized by a power spectral density that is proportional to *f*^−*γ*^ where *f* is the frequency and 0.7 < *γ* < 1.3 [48]. The flicker noise leads to a degradation of the SNR that is more important for devices having smaller gate areas [49].

In what follows, we characterize the noise at different bias points for three SiNRs, ranging from 110 nm to 10 µm in width, by performing the fast Fourier transform (FFT) [50] of *I*_D-S_ sampled at *f*_S_ = 10 Hz. A spectral filtering technique [51] has been employed to compensate for the distortions caused by aliasing, which can impact the scaling exponents of *f*^−*γ*^ noises (see Supplementary Figure S6) [51,52,53,54].

**Figure 5:**
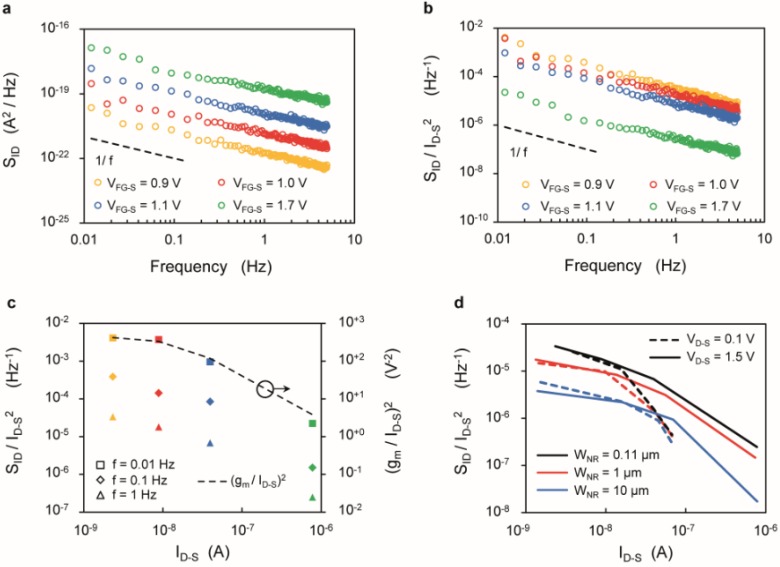
(**a**–**c**) Noise characteristics of a SiNR with length *L*_NR_ = 2375 nm, width *W*_NR_ = 110 nm and drain voltage *V*_D-S_ = 1.5 V. (**a**) Low-frequency drain current noise spectral density (*S*_ID_) characteristics; (**b**) *S*_ID_/*I*_D-S_^2^ plotted against frequency; (**c**) *S*_ID_/*I*_D-S_^2^ plotted for different frequencies (*f* = 0.01 Hz, *f* = 0.1 Hz and *f* = 1 Hz) as a function of *I*_D-S_, compared with the (*g*_m_/*I*_D‑S_)^2^ ratio (dotted line, right axis); (**d**) *S*_ID_/*I*_D-S_^2^ plotted as a function of *I*_D-S_ for SiNRs with same length (*L*_NR_ = 2375 nm) and different widths (*W*_NR_ = 110 nm, *W*_NR_ = 1 µm and *W*_NR_ = 10 µm) biased to work in saturation (*V*_D-S_ = 1.5 V, solid lines) and linear regime (*V*_D-S_ = 0.1 V, dotted lines), at a frequency *f* = 1 Hz.

Figure 5a shows the current noise power spectral density (*S*_ID_) for a SiNR (*L*_NR_ = 2375 nm, *W*_NR_ = 110 nm) biased in subthreshold (*V*_FG-S_ = 0.9 V), weak inversion (*V*_FG-S_ = 1.0 V and 1.1 V) and above threshold (*V*_FG-S_ = 1.7 V), respectively. The *S*_ID_ of the silicon nanoribbon follows the expected 1/*f* behavior at low frequency, with larger noise at high *V*_FG-S_.

On the other hand, Figure 5b shows that when considering the ratio between noise power and squared signal, working above threshold leads to higher SNR. In support of this observation, Figure 5c reports *S*_ID_/*I*_D-S_^2^ *vs*. *I*_D-S_ for different sampling frequencies, showing that working at larger currents lead to smaller noise-to-signal ratio. These results are consistent with previously reported works [55], in which the SNR is maximized at the peak transconductance. Moreover, the plot shows that the quantity *S*_ID_/*I*_D-S_^2^ and (*g*_m_/*I*_D-S_)^2^ follow the same trend *vs.* *I*_D-S_ (see also Supplementary Figure S7), thus supporting the validity of the carrier-number fluctuation (Δ*N*) arising from the dynamic trapping and de-trapping of free carriers within the gate oxide as the dominating factor of the flicker noise [48,49].

Finally, Figure 5d compares the three SiNRs with different widths in terms of the *S*_ID_/*I*_D‑S_^2^ factor. In the low current region, the SNR depends only weakly on the drain voltage. Instead, in strong inversion, the saturation regime ensures a lower noise-to-signal ratio.

In conclusion, since nano-sized transistors occupying smaller areas suffer more from intrinsic noise, those approaches aimed at increasing the array density of a chip by employing tinier pH sensors must take into account the degradation of the SNR, which would ultimately negatively affect the resolution in pH. In what follows, we show that multi-wire transistors can reduce this limit and thus allow smaller area per sensing pixel with improved resolution.

### 3.3. Multiple-Wire Devices

In this section, we show that devices consisting of multiple nanowires connected in parallel exceed the performance of single devices occupying the same footprint area, and we compare their performances with existing planar and tri-gate devices. In fact, multi-wires devices benefit, on one hand, from the enhanced sensitivity of narrower silicon structures and, on the other hand, from the lower noise provided by their larger interface area [56]. Moreover, previous works have shown the superior inter-device uniformity of multi-wire devices when compared to single SiNR [57]. As a consequence, multi-wire devices alleviate the variability of electrical parameters such as *V*_TH_, SS and *g*_m_.

**Figure 6:**
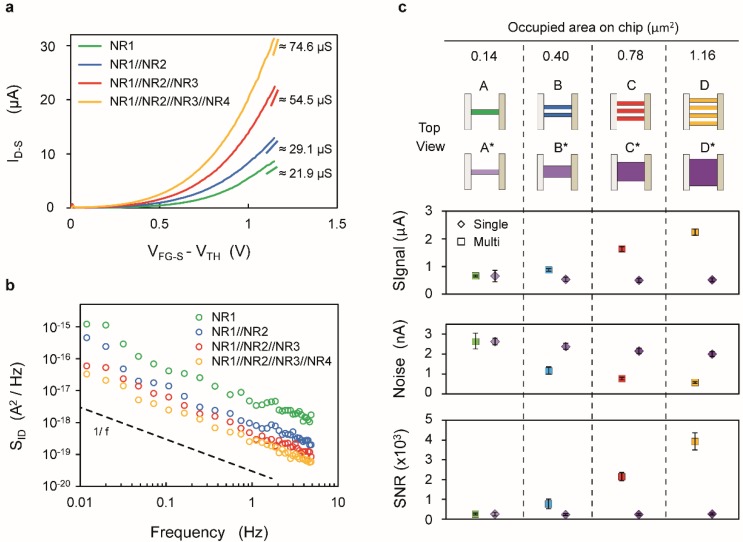
(**a**–**c**) The SiNRs have dimensions as follows: *W*_NR1,2_ = 60 nm, *W*_NR3,4_ = 110 nm with *L*_NR1,2,3,4_ = 2375 nm. (**a**) Drain current (*I*_D‑S_) *vs.* overdrive voltage (*V*_FG-S_ − V_TH_) characteristics; the value of the transconductance extracted at *V*_FG‑S_ − *V*_TH_ = 1.1 V is indicated on the right; (**b**) Low-frequency current noise spectral density (*S*_ID_) characteristics (**c**) Signal, rms noise (as obtained by integrating S_ID_ over 1Hz bandwidth centered around 1Hz) and SNR values for multi-finger devices (□) and for single SiNR occupying the same area on chip (◊) with color-coded cartoon representation of the devices with the indication of the occupied area, considering a distance of 50 nm between fingers.

The four common-source devices characterized in this work exhibit independent drain contacts for each finger, which allows us to compare the performance of the single-wire with that of two-, three- and four-wire devices (Figure 6, cartoon A, B, C and D respectively). The devices were biased above threshold (*V*_FG‑S_ − *V*_TH_ ≈ 1 V) and in saturation regime (*V*_D‑S_ = 1.5 V). The results show an increase of the maximum transconductance with the number of SiNR fingers (Figure 6a). However, these values are smaller than the sum of the transconductance of individual wires, mainly due to the slightly different threshold voltage of the considered four wires, which results in a degradation of the average *g_m_*. As shown in Figure 4c, the current sensitivity is directly linked to the transconductance of the device and, therefore, it also increases with the number of fingers.

Figure 6b reports the current noise as a function of the frequency for devices with increasing number of fingers showing an improvement for devices featuring larger area, as expected from *S*_ID_ scaling rules. As for Figure 5, the spectra have been compensated for aliasing distortions (Figure S6, Supplementary). Figure 6c reports the Signal, Noise and SNR for devices of increasing occupied area featuring multiple nanowires (□). The Signal value corresponds to the measured *g*_m_ of the device multiplied by Δ*V*_TH_/ΔpH (assumed to be 30 mV/pH, according to Figure 4b) and the related error bars are defined based on the variability over the timescale of an experiment (a few hours). The Noise value of multi-wire devices is obtained by calculating the square root of the integral of the noise spectrum *S*_ID_ in 1 Hz bandwidth centered at 1 Hz. The corresponding error bars are calculated from the prediction bounds of the interpolated spectrum (Figure S8, Supplementary).

The analysis of sensitivity and noise characteristics allows us to evaluate the resolution of the devices, *i.e.*, the smallest detectable pH change. The resolution (δpH) is calculated as pH change giving a signal-to-noise ratio SNR = 3:

(1) $$SNR=\;\frac{\Delta I_{D-S}/\;\Delta pH\;}{\sqrt{\int^{\text{​}}S_{ID}df}}\;=3$$

Since in Figure 4c we see that Δ*I*_DS_/ΔpH = g_m_ Δ*V*_TH_/ΔpH, we can also compute

(2) $$SNR=\;g_{m}\frac{\Delta V_{TH}/\;\Delta pH\;}{\sqrt{\int^{\text{​}}S_{ID}df}}=\frac{\Delta V_{TH}/\;\Delta pH\;}{\sqrt{\int^{\text{​}}S_{V}df}}$$

where $S_{V}=S_{ID}/g_{m}^{2}$ is the voltage noise power spectral density. The noise-equivalent pH value is obtained by dividing the noise level (square root of the integral of the current noise spectra over the measurement frequency bandwidth) by the current sensitivity (Δ*I*_D‑S_/ΔpH). A resolution of ~0.8‰ of pH change is achieved in a 1 Hz bandwidth, centered at 1 Hz, by a four-fingers device occupying approximately 1.16 µm^2^. In terms of the lowest detectable change of electron surface density at the gate interface, the achieved pH resolution corresponds to 1.78 × 10^−17^ C/µm^2^.

This result is close to the lowest reported value obtained with tri-gate nanowires (0.5‰ of a pH shift in a 1 Hz bandwidth centered at 10 Hz) employing a device with a 7 µm^2^ footprint [25]. Nevertheless, in the context of ever increasing array density of integrated pH sensors, the most indicative parameter is the resolution that can be achieved in a unit of occupied footprint area on the chip. In this regard, our results outperform the previous works (0.0008 pH *vs.* 0.0005·√(7) = 0.0013 pH change with 1 µm^2^ footprint device) [25], despite our more conservative choice of the SNR level to define the resolution (SNR = 3) and of a lower central frequency (*f*_C_) for the bandwidth (1 Hz *vs.* 10 Hz). When evaluated under the same conditions (SNR = 1, *f*_C_ = 10 Hz), our system outperforms the state-of-the-art by two orders of magnitude.

In order to compare the performance of multi-wire design with single nanoribbon devices, the trend of single SiNRs of equivalent area (A*, B*, C* and D*) has been reported in Figure 6c (◊). In this case, the Signal values have been derived from Figure 4d, with the error bars taking into account the interpolation error of the fitted trend. The Noise values (◊ Noise plot Figure 6c) are evaluated on the basis of the noise characterization of single ribbon devices (Figure 5d, *V*_D‑S_ = 1.5 V) and the corresponding error bars take into account the interpolation error (Figure S9, Supplementary). This suggests that the SNR of multi-wire devices increases with the area with a steeper trend than single nanoribbons and, in particular, a device occupying 1.16 µm^2^ is characterized by a SNR that is 15 times higher than the one of a single nanoribbon of identical footprint area.

Clearly, the resolution could be further improved by increasing the density of interconnected nanowires per device, and by sampling at higher frequencies [58]. Moreover, the employment of high-*k* gate dielectrics such as Al_2_O_3_ and HfO_2_ [16,31] would induce a larger change of surface potential upon a pH change, leading to a higher signal transduced by the sensor.

## 4. Conclusions

The performances including an improved sensitivity of nano-sized tri-dimensional silicon devices as detectors of surface charge in electrolyte environment have been investigated. The tri-gate configuration gives silicon nanoribbons undeniable advantages with respect to planar devices and, in particular, in this work we show that ribbons with width below a few hundreds of nanometers show enhanced conductivity properties, ultimately leading to higher sensitivity.

The enhanced performance characteristic of narrower wires can be combined with the lower noise granted by the larger gate area of multiple nanowires connected in parallel. Moreover, multi-wire devices assure improved inter-device uniformity in terms of the conductance characteristics.

In conclusion, we have shown that tri-dimensional, multi-wire devices constitute a successful approach to scale down the size of the single pH sensor, achieving unprecedented pH resolution per unit occupied footprint area. This feature is of particular interest in integrated pH-based bioanalytics on CMOS technology such as DNA sequencing and quantitative PCR, which lean toward sensor arrays of ever-increasing density. Our technology relies on highly-controlled top-down CMOS-compatible fabrication processes, which are required in large-scale integration.

## Supplementary Files

Supplementary File 1
