# Supplementary material for: Multi-Wire Tri-Gate Silicon Nanowires Reaching Milli-pH Unit Resolution in One Micron Square Footprint
Source: Biosensors (Basel). 2016 Mar 15;6(1):9. doi: 10.3390/bios6010009 (PMC4810401; doi:10.3390/bios6010009)
Supplement: Supplementary File 1 [file biosensors-06-00009-s001.pdf]

Supplementary Materials: Multi-Wire Tri-Gate Silicon Nanowires Reaching Milli-Units pH Resolution in One Micron Square Footprint

Enrico Accastelli, Paolo Scarbolo, Thomas Ernst, Pierpaolo Palestri, Luca Selmi and Carlotta Guiducci

Modeling of pH Sensing

According to the site-binding model applied to ISFETs [1–3], solutions in contact with the gate oxide induce a pH-dependent surface potential. In particular, the surface of the front-gate oxide becomes negatively charged when in contact with electrolyte solutions with pH values larger than its isoelectric point (pI ≈ 2 in the case of SiO_2). This phenomenon is due to the deprotonation (protonation) of the silanol groups in the presence of OH^- (H^+) ions in the electrolyte solution. The accumulated charge creates a surface potential that affects the SiNR conductance in case of a fixed bias. The pH-induced change of the electrical current ($\partial I_{D\text{-}S}/\partial$pH) can be expressed as:

$$\frac{\partial I_{\mathrm{D-S}}}{\partial \mathrm{pH}} = \left[\frac{\partial I_{\mathrm{D-S}}}{\partial (V_{\mathrm{G-S}} - V_{\mathrm{TH}})}\right]\left[\frac{\partial (V_{\mathrm{G-S}} - V_{\mathrm{TH}})}{\partial \Psi_0}\right]\left(\frac{\partial \Psi_0}{\partial \mathrm{pH}}\right) = A \cdot B \cdot C \quad (1)$$

where $V_{G\text{-}S}$ denotes the gate-source voltage, V_{TH} the threshold voltage, and ψ_0 the potential at the oxide/electrolyte interface. Term *A* in Equation (1) represents the trans-conductance of the FET. Term *B* can be expressed as follows:

$$\frac{\partial (V_{\mathrm{G-S}} - V_{\mathrm{TH}})}{\partial \Psi_0} = \frac{\partial (V_{\mathrm{G-S}} - V_{\mathrm{TH}})}{\partial Q_{\mathrm{i}}} \cdot \frac{\partial Q_{\mathrm{i}}}{\partial \Psi_0} = C_{\mathrm{GC}}{}^{-1}\, C_{\mathrm{EC}} \quad (2)$$

where C_{GC} represents the capacitance between the gate ($V_{G\text{-}S}$) and the inversion charge in the channel (Q_i) and C_{EC} relates the induced surface potential with the inversion density. Both term *A* and term *B* depend on the electrical properties of the device and on the bias configuration. In case of pH sensing, in which the detected charge is located directly on the front-gate oxide, C_{EC} is the gate oxide capacitance C_{OX} and, therefore, it depends on the material the gate insulation is made of and its thickness.

The term C of Equation (1) is given by the Nernst's equation:

$$\frac{\partial \Psi_0}{\partial \mathrm{pH}} = -2.303 \frac{k_B T}{q} \alpha \quad (3.1)$$

$$\alpha = \frac{1}{1 + \frac{C_{\mathrm{EDL}}}{C_{\mathrm{B}}}} \quad (3.2)$$

where k_B denotes the Boltzmann constant, *T* the absolute temperature, *q* the elementary charge, α the dimensionless sensitivity parameter ($0 \leq \alpha \leq 1$), with C_{EDL} indicating the electrical double layer (EDL) capacitance and C_B the buffer capacitance of the surface determined by the density of active OH groups on the gate oxide. The ISFET sensitivity reaches the ideal Nernst limit (59.5 mV/pH at $T = 300$ K) when $\alpha = 1$, *i.e.*, when C_B is much larger than C_{EDL}. This is the case, e.g., for Al_2O_3 and HfO_2 front-gate oxides [4,5].

Indeed the device sensitivity to pH depends not only on the gate oxide material, but also on the bias of the device and the buffer conditions. As previously reported [5,6], in the case of back-gating ($V_{G\text{-}S} \equiv V_{BG\text{-}S}$) one can achieve values of $\Delta V_{TH}/\Delta$pH beyond the Nernst's limit. The amplification factor comes from Equation (2) that simplifies as follows:

$$\left[\frac{\partial (V_{\mathrm{BG-S}} - V_{\mathrm{TH}})}{\partial Q_{\mathrm{i}}} \cdot \frac{\partial Q_{\mathrm{i}}}{\partial \Psi_0}\right] \approx (C_{\mathrm{BOX}})^{-1}\, C_{\mathrm{OX}} > 1 \quad (4)$$

where C_{BOX} denotes the capacitance of the bulk oxide. The change in surface potential on the front-gate side translates effectively into a larger back-gate threshold voltage shift ($\Delta V_{TH,BG} > \Delta\psi_0$), due to the difference between the top and back oxide thicknesses [5,6]. In case of front-gating, instead, there is no amplification factor as term *B* of Equation (1) is reduced to 1.

This amplification factor can also be tuned by changing the location and number of active gates. As reported by Jae-Hyuk *et al.*[7], by designing two active lateral gates, the capacitance ratio given by Equation (2) increases proportionally with the width of the device and so does the sensitivity.

To summarize, an effective strategy to maximize the pH sensitivity in terms of $\Delta V_{TH}/\Delta pH$, consists in maximizing the asymmetry between the active gate biasing the device and the biochemical interaction occurring at the top oxide surface, by biasing the device with a back gate, or, in the case of SiO_2 surfaces, by utilizing solutions with lower ionic strength. It is important to remark, however, that the back-gating configuration with electrolyte solution left electrically floating can lead to instability problems [8,9].

Figure S1. Biasing Point

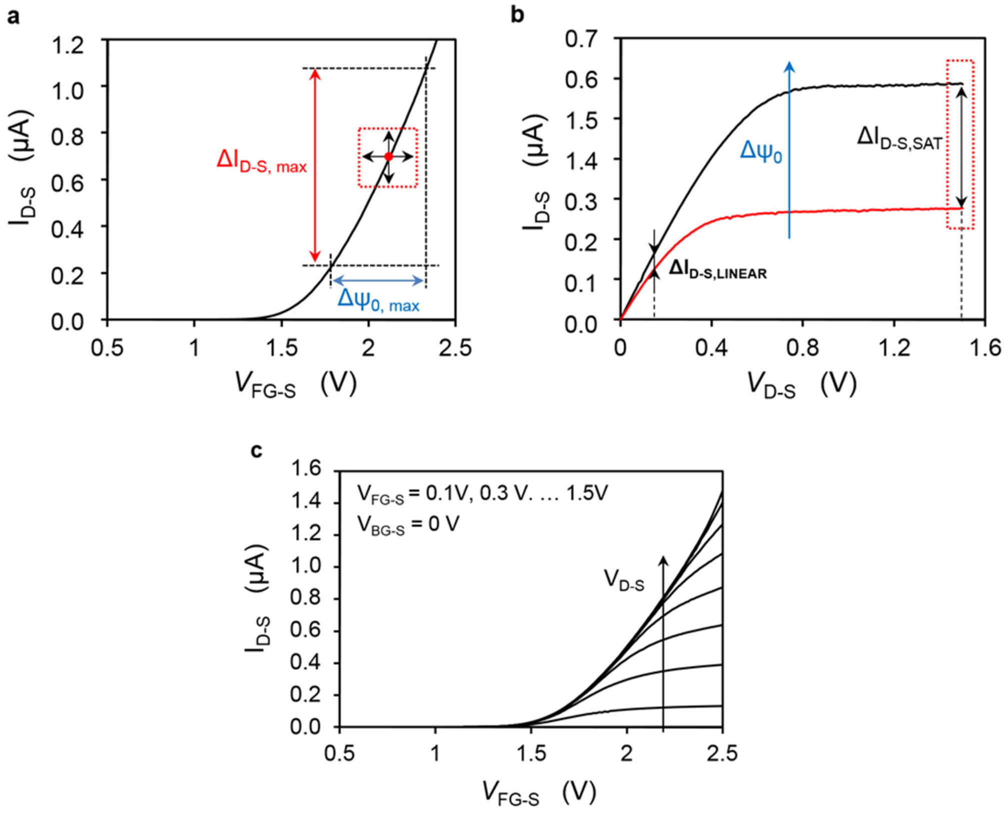

Figure S1. (**a**) Drain current ($I_{D\text{-}S}$) *versus* gate voltage ($V_{FG\text{-}S}$); (**b**) Drain current ($I_{D\text{-}S}$) *versus* drain voltage ($V_{D\text{-}S}$). The red dashed boxes enclose the working region of interest; (**c**) Drain current ($I_{D\text{-}S}$) *versus* gate voltage ($V_{FG\text{-}S}$).

The SiNRs were biased to operate above threshold ($V_{FG\text{-}S}$ ~ 1.8 V), as it has been experimentally observed that this condition guarantees improved performances in terms of drain current stability over time. Indeed subthreshold conditions assure higher sensitivity in terms of relative change of the current with the surface potential, while above threshold there is a higher sensitivity in terms of absolute change ($\Delta I_{D\text{-}S}/\Delta pH$) of the current and a more linear response. In particular, the gate voltage was set so that the maximum variation in surface potential connected to a change in pH does not shift the FET operating point out of the chosen region of operation (Figure S1a).

In order to maximize the absolute change of the drain current with the pH, the devices are biased in saturation ($V_{D\text{-}S}$ = 1.5 V) (Figure S1b). Moreover, a high $V_{D\text{-}S}$ maximizes the dynamic range of the above threshold region of operation (Figure S1c).

Figure S2. Hysteresis between the Forward and Backward pH Sweep

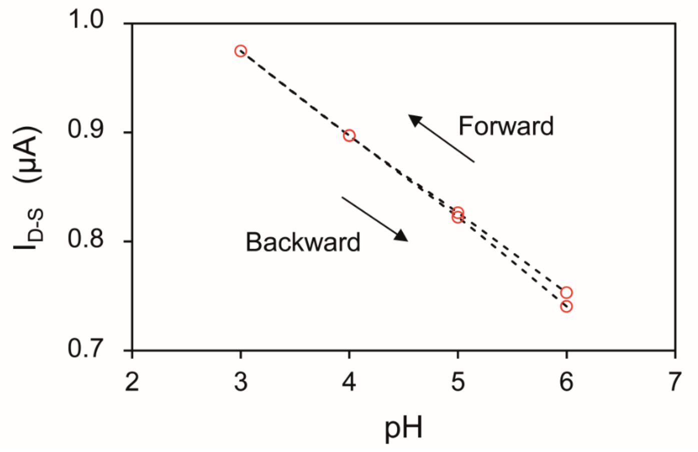

Figure S2. Drain current (I_{D-S}) observed at fixed voltage bias upon injection of decreasing/increasing series of pH values (Figure 3a). The forward and backward sweeps show negligible hysteresis.

Figure S3. Conditioning of the SiO2 Surface

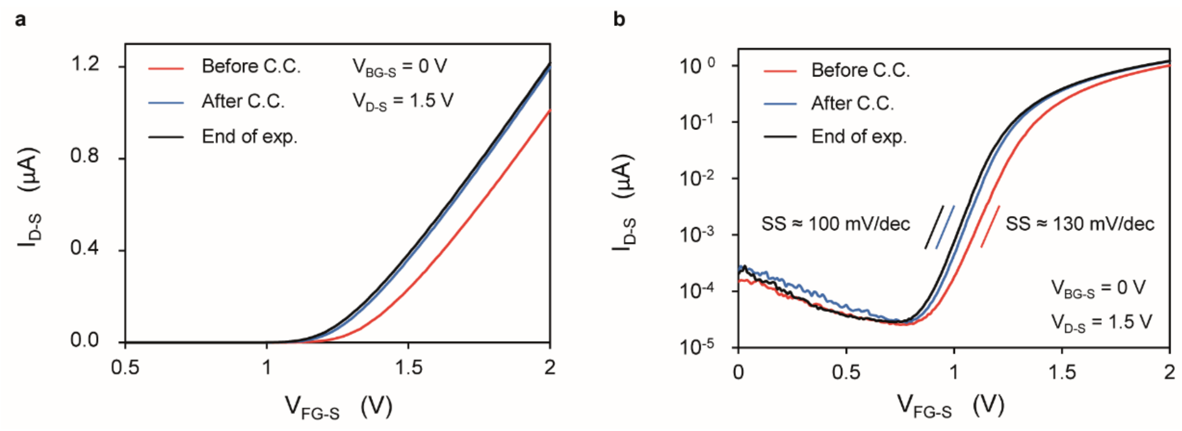

Figure S3. Drain current (I_{D-S}) *versus* gate voltage (V_{FG-S}) characteristics plotted in (**a**) linear and (**b**) logarithmic scale, measured before the conditioning cycle (red line), after the conditioning cycle (blue line) and at the end of experiment (black line).

A conditioning cycle is performed in order to prime the front-gate oxide surface. The conditioning cycle consists in injecting the 10 µM KCl buffer solution until the current stabilizes, followed by a solution with pH 3 and pH 8, which are separated by a 10 µM KCl washing step. This conditioning of the surface improves the electrical characteristics of the device, e.g., the subthreshold swing (*SS*) stabilizes from 130 mV/dec to 100 mV/dec and then remains constant throughout the whole experiment. Once conditioned, the device I_{D-S}-V_{FG-S} characteristic only shifts with a change of the potential at the oxide/electrolyte interface.

Figure S4. Influence of Ionic Strength

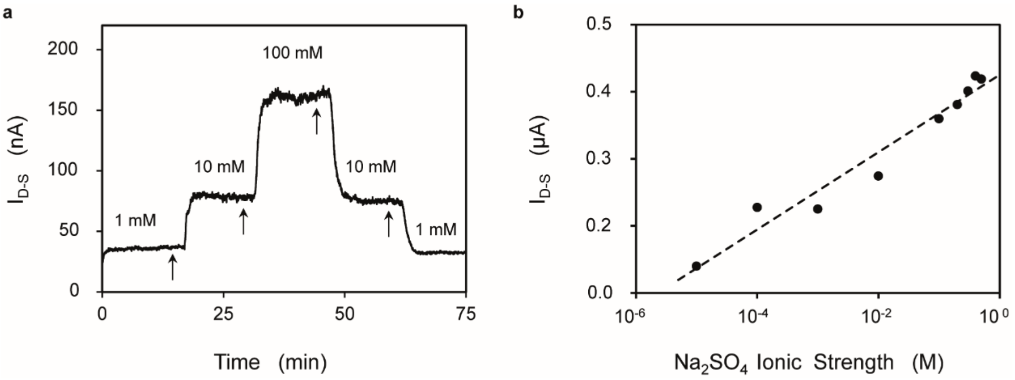

Figure S4. (**a**) Real-time drain current ($I_{D\text{-}S}$) measurement upon injection of solutions with different ionic strength values and a constant pH of 6. The arrows indicate the injection of the solution (delays due to tubing have been considered); (**b**) Drain current ($I_{D\text{-}S}$) *versus* Na_2SO_4 ionic strength, showing a logarithmic relationship.

The silica surface exposed to the solution charges up and attracts a layer of counter ions to maintain the overall charge neutrality, forming an electrical double layer (EDL). The capacitance of the EDL depends on the concentration of the electrolyte and affects the drain current. In order to verify this fact, we put solutions with increasing concentration of Na_2SO_4 in contact with the SiNRs and monitor the change of the drain current (Figure S4a). As shown in Figure S4b, the current increases logarithmically for increasing ionic strength of the solution.

To address this issue, the solutions with different pH were designed to minimize the differences in the total ionic strength.

Figure S5. Extraction of Series Resistances and Compensation Procedure

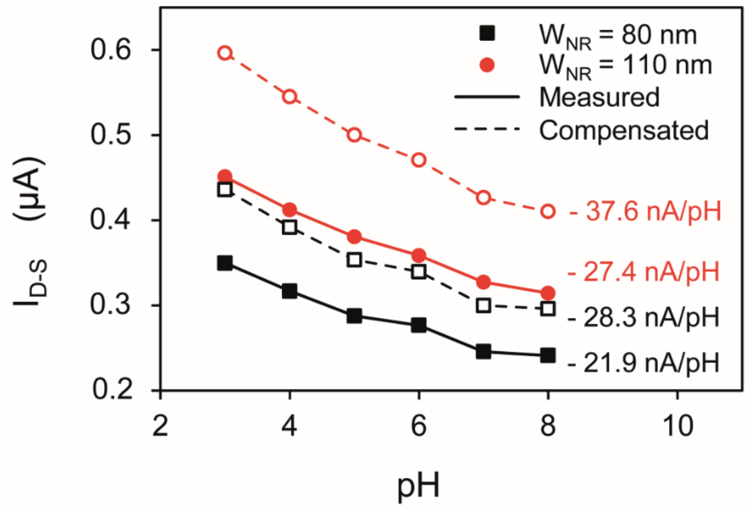

Figure S5. Drain current ($I_{D\text{-}S}$) plotted against the pH, for two devices with same length (L_{NR} = 2375 nm) and different widths (W_{NR} = 80 nm and 110 nm), before and after compensation for the series resistances. The correspondent current sensitivity is indicated.

The SiNRs feature a relatively big series resistance that was extracted by measuring the $I_{D\text{-}S}$-$V_{G\text{-}S}$ in the linear region, for 2 different $V_{D\text{-}S}$ (*i.e.*, $V_{D\text{-}S}$ = 100 mV and $V_{D\text{-}S}$ = 200 mV) [10]. The drain (R_D) and source (R_S) series resistances are computed from the ratio between the two measured currents $I_{D\text{-}S,\ 200\ mV}/I_{D\text{-}S,\ 100\ mV}$. In particular, the saturated part of the $I_{D\text{-}S}/V_{FG\text{-}S}$ curve is considered, where the series resistances are comparable with the channel resistance.

The $I_{D\text{-}S}$ in linear regime ($V_{D\text{-}S} \leq V_{G\text{-}S} - V_{TH}$) can be expressed as:

$$I_{D-S} = K(V_{G-S} - V_{TH} - 0.5 \cdot V'_{D-S})V'_{D-S} \quad (5)$$

where $K = \mu_{EFF}C_{OX}W_{NR}/L_{NR}$ and μ_{EFF} indicates the effective carrier mobility. However, due to the series resistances, the effective gate (V'_{FG-S}) and drain voltage (V'_{D-S}) can be defined as

$$V'_{G-S} = V_{G-S} - R_S I_{D-S} \quad (6.1)$$

$$V'_{D-S} = V_{D-S} - (R_S + R_D)I_{D-S} \quad (6.2)$$

must be considered in Equation (5), which becomes:

$$I_{D-S} = K\left[V_{G-S} - R_S I_{D-S} - V_{TH} - \frac{V_{D-S} - (R_S + R_D)I_{D-S}}{2}\right][V_{D-S} - (R_S + R_D)I_{D-S}] \quad (7)$$

Since the device layout is not symmetric, $R_S \neq R_D$. If we write $R_S = \beta R_D$, with β indicating the ration between the length of source and drain leads; Equation (7) becomes:

$$I_{D-S} = K\left[V_{G-S} - V_{TH} - \frac{V_{D-S}}{2} + \frac{(1-\beta)R_D I_{D-S}}{2}\right][V_{D-S} - (1+\beta)R_D I_{D-S}] \quad (8)$$

By calculating the ratio $I_{D-S,\,200\,mV}/I_{D-S,\,100\,mV}$ the dependence on K factor is removed and we obtain an equation with the only variable R_D. Once the values of R_D and R_S are known, it is possible to compensate for their effect and the corrected I_{D-S}-V_{FG-S} obtained.

In this work, the devices operates in the saturation working region ($V_{D-S} = 1.5$ V $> V_{FG-S} - V_{TH}$). The I_{D-S} in this working regime can be expressed as:

$$I_{D-S} = 0.5 \cdot K(V'_{G-S} - V_{TH})^2 \quad (9)$$

Taking into account the series resistances, Equation (9) becomes:

$$I_{D-S} = K(V_{G-S} - R_S I_{D-S} - V_{TH})^2 \quad (10)$$

From Equation (10) the term K can be extracted and substituted in

$$I'_{D-S} = 0.5 \cdot K(V_{G-S} - V_{TH})^2 \quad (11)$$

where we denote the compensated drain current as I'_{D-S}, which can therefore be expressed as:

$$I'_{D-S} = \frac{I_{D-S}}{(V_{FG-S} - V_{TH} - R_S I_{D-S})^2}(V_{G-S} - V_{TH})^2 \quad (12)$$

Figure S5 shows the result of the compensation procedure on the current sensitivity of 2 SiNRs.

Figure S6. Anti-Aliasing Spectral Filtering

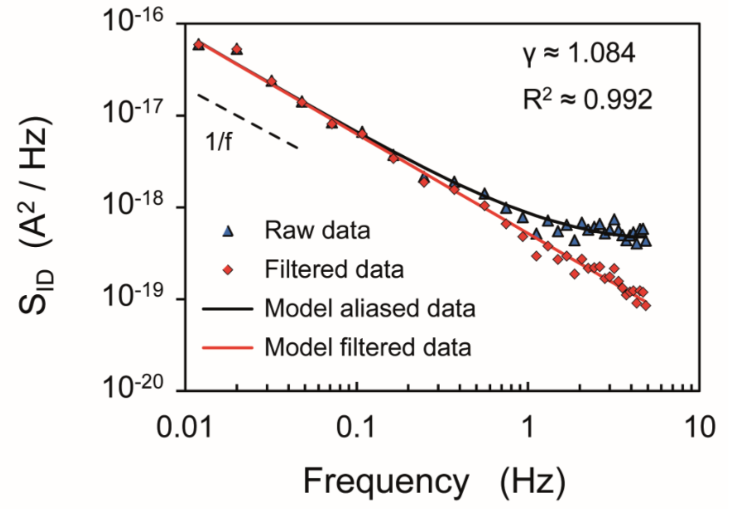

Figure S6. Effect of the employed spectral filtering technique on a set of raw data.

The spectral filtering technique reported in the work of Kirchner *et al.* [11] has been employed to compensate for the distortions caused by aliasing, which can impact the scaling exponent of $f^{-\gamma}$ noise [11–14]. The scaling factor γ is commonly estimated from the power-law slope of the FFT of the noise time-series. However, as these noises typically have significant power above the Nyquist frequency, measurements of their power spectra will often be severely distorted by aliasing. A spectral filtering technique is employed to correct the distortions introduced by spectral aliasing, and recovers the broadband spectrum of $f^{-\gamma}$ noises. Further details on the employed filtering method can be found in the work of Kirchner *et al.*[11].

Estimating the alias filter requires a model for the spectrum, so that the ratio of the signal power and the signal-plus-alias power can be estimated. The employed spectral model $S_{\text{model}}(f)$ for the noise is:

$$S_{\text{model}}(f) = \frac{S_0 f^{-\gamma}}{1 + (f/f_{\text{BANDWIDTH}})^2} \tag{13}$$

This model spectrum scales as $f^{-\gamma}$ below some specified boundary frequency $f_{\text{BANDWIDTH}}$, then rolls over to a steeper spectral slope at higher frequencies. The frequency $f_{\text{BANDWIDTH}}$ indicates the bandwidth of the measurement system, which can be assumed to be not larger than ~50 kHz.

Figure S6 illustrates the effect of the filtering technique on the aliased-affected noise spectrum. The iterative algorithm evaluates the value of γ for which the sum of the noise spectrum, modeled as reported in Equation (13), and the corresponding alias best fits the experimental data. At the end of the iterative procedure, the obtained value of γ is ≈1, confirming the nature of the noise affecting the nanoribbon (flicker noise).

Since $f_{\text{BANDWIDTH}} >> f_S = 10$ Hz, the exact value of $f_{\text{BANDWIDTH}}$ has little effect on the alias-filtered spectrum. For example, changing the value of $f_{\text{BANDWIDTH}}$ in the range 1 kHz–1 MHz introduces a discrepancy of less than 1% in the estimation of the scaling factor γ.

Moreover, the spectral filter is conceived so as to remove the (proportional) effect of the modeled aliasing, rather than forcing the alias-filtered spectrum to conform to the model spectrum. As a consequence, mis-specification of the model spectrum has only a small effect on the results of the alias filtering procedure.

The white noise current spectral density of MOSFET transistor can be estimated as [15]:

$$4K_B T \cdot 2/3 \cdot g_m \approx 10^{-25}\ \text{A/Hz}^2 \tag{14}$$

Since at the considered band the level of the white noise is more than 5 orders of magnitude smaller than the $f^{-\gamma}$ noise introduced by the nanoribbon, its impact on the aliasing is negligible.

Figure S7. Noise Characterization in Linear Regime

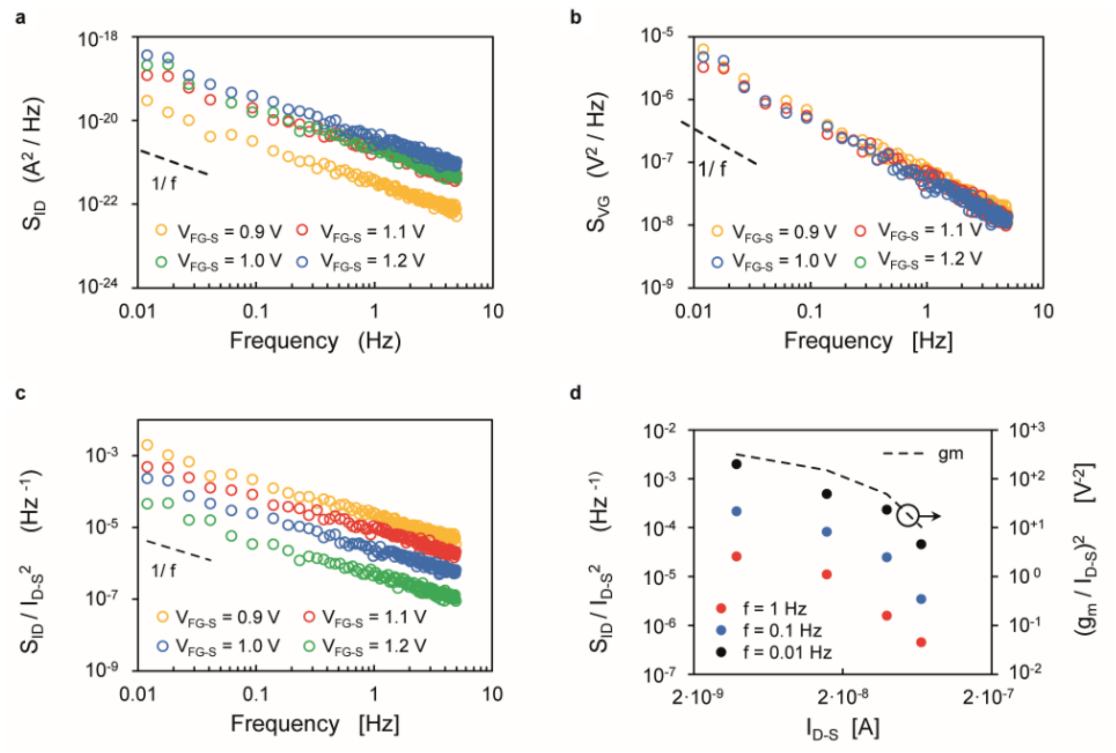

Figure S7. (**a**–**d**) Noise characteristics of a SiNR (L_{NR} = 2375 nm, W_{NR} = 110 nm) for different gate voltages ($V_{FG\text{-}S}$) and a $V_{D\text{-}S}$ of 0.1 V. (**a**) Low-frequency drain current noise spectral density (S_{ID}) characteristics; (**b**) Low-frequency gate voltage noise spectral density (S_{VG}) characteristics; (**c**) $S_{ID}/I_{D\text{-}S}^2$ plotted against frequency; (**d**) $S_{ID}/I_{D\text{-}S}^2$ plotted for different frequencies (f = 0.01 Hz, f = 0.1 Hz and f = 1 Hz) as a function of $I_{D\text{-}S}$, compared with the $(g_m/I_{D\text{-}S})^2$ ratio (dotted line, right axis).

Figure S7a shows the current noise power spectral density (S_{ID}) characteristics of a SiNR (L_{NR} = 2375 nm, W_{NR} = 110 nm) at a fixed $V_{D\text{-}S}$ of 0.1 V and increasing $V_{FG\text{-}S}$ that polarizes the device to work in subthreshold ($V_{FG\text{-}S}$ = 0.9 V), weak ($V_{FG\text{-}S}$ = 1.0 V and 1.1 V) and strong inversion ($V_{FG\text{-}S}$ = 1.2 V). The S_{ID} of the SiNR, compensated for the aliasing, follows a $1/f$ behavior. Contrary to what happens in saturation regime ($V_{D\text{-}S}$ = 1.5 V), the S_{ID} of the SiNR does not increase with higher voltages, but, instead, appears to stabilize. The same behavior can be seen in the gate voltage noise power spectral density (S_{VG}), in which the noise level only slightly depends on the applied $V_{FG\text{-}S}$, with even lower noise level for higher voltages (Figure S7b). It is worth noting that according to [16] the S_{VG} spectrum can be expressed as:

$$S_{VG}|_{fo} = \frac{S_{ID}|_{fo}}{g_m{}^2} = N_{OT} \cdot \frac{q^2}{C_{OX}{}^2 \cdot W_{NR} \cdot L_{NR} \cdot f_0} \quad (15)$$

where f_0 represents the band at which S_{VG} is evaluated. From Equation (15) and considering a band f_0 of 1 Hz with a central frequency of 1 Hz, it is possible to estimate the density of traps N_{OT}, which in the case of the device of Figure S7 results to be approximately $1.12 \cdot 10^{10}$ cm^{-2}, which is somewhat larger than similar reports in literature but not unphysical.

Using instead the equation proposed in [17]:

$$S_{VG} = N_{OT} \cdot \frac{kT \cdot q^2}{\alpha_t \cdot C_{OX}{}^2 \cdot W_{NR} \cdot L_{NR} \cdot f_0} \quad (16)$$

where $1/\alpha_t$ = 0.1 nm is the tunneling length, we obtain $N_{OT} = 4.3 \cdot 10^{19}$ cm^{-3}eV^{-1}. This value is on the high side of those reported in literature, but not unphysical, given the very large value chosen for α_t.

By plotting $S_{ID}/I_{D\text{-}S}^2$ against the frequency (Figure S7c), it is even more clear that, from an SNR perspective, it is convenient to work in strong inversion, at high voltages ($V_{FG\text{-}S}$ = 1.2 V). The same conclusion can be drawn when plotting the $S_{ID}/I_{D\text{-}S}^2$ *vs.* $I_{D\text{-}S}$ for different sample frequencies (Figure S7d). Moreover, the fact that $S_{ID}/I_{D\text{-}S}^2$ *vs.* $I_{D\text{-}S}$ curves run parallel to the $(g_m/I_{D\text{-}S})^2$ *vs.* $I_{D\text{-}S}$, when plotted in comparable scales, supports the validity of the carrier number fluctuations (ΔN) as the dominating factor of the flicker noise [18,19].

Figure S8. Evaluation of the Noise Levels of Multi-Wire Devices

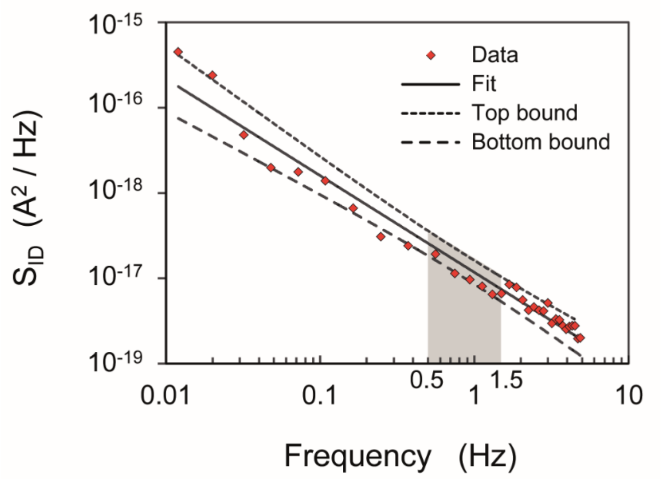

Figure S8. Fitting of the noise data compensated for aliasing. The noise value, top error bar and bottom error bars are obtained considering a bandwidth of interest (0.5 Hz–1.5 Hz) and calculating the integrals of the fit, the top bound and bottom bound lines, respectively.

The noise values of Figure 6c for multi-wire devices are obtained by fitting the data compensated for the aliasing. In particular, the noise level is obtained by calculating the square root of the integral of S_{ID} in the frequency range of interest (0.5 Hz–1.5 Hz). The error bars are calculated from the integrals of the prediction bounds, determined with 99.999% confidence.

Figure S9. Evaluation of the Noise Levels of Single-Wire Devices

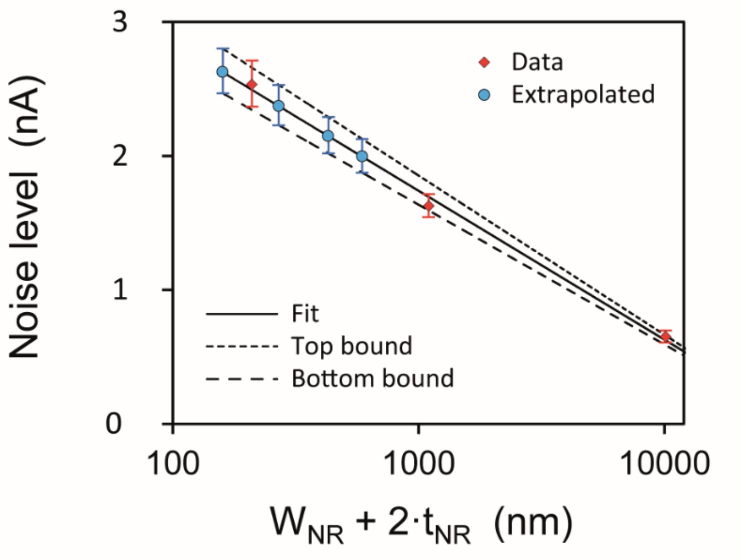

Figure S9. Evaluation of the noise levels of single-wire devices.

The noise values of single-ribbons are obtained by fitting the noise dependence of the single-ribbons of Figure 5d on the device cross-section total area ($W_{NR} + 2 \cdot t_{NR}$). The noise values and the corresponding error bars of the devices of Figure 5d are calculated as reported Figure S8. The error bars of the extrapolated data are calculated as the difference between the fit and the top/bottom bounds as shown in Figure S9.

Figure S10. Ag/AgCl Reference Electrode

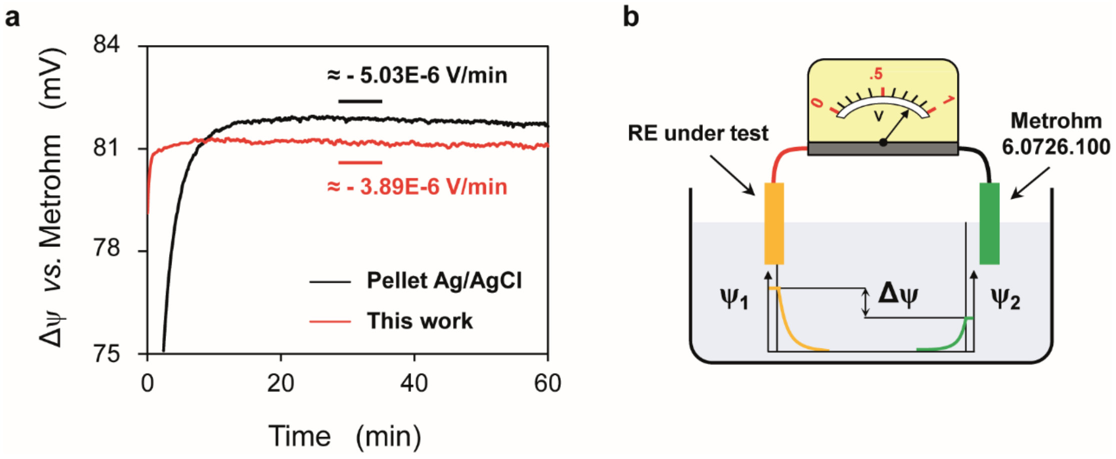

Figure S10. (**a**) Comparison of the potential drift over time in the case of fabricated (red line) and commercial (black line) RE; (**b**) Cartoon representation of the employed measurement configuration necessary to evaluate the RE voltage drift.

In order to evaluate the performances of the home-made Ag/AgCl pseudo-reference electrodes (REs), we compared the corresponding voltage drift over time of the RE and the one of a commercial Ag/AgCl (E255 pellet, PHYMEP). The drifts are measured *vs.* a double junction Ag/AgCl, (6.0726.100, Metrohm) at a concentration of 3 M (Figure S10).

References

1. Bousse, L.; Bergveld, P. The Role of Buried Oh-Sites in the Response Mechanism of Inorganic-Gate Ph-Sensitive Isfets. *Sens. Actuators* **1984**, *6*, 65–78.
2. Bergveld, P.; VanHal, R.; Eijkel, J. The Remarkable Similarity Between the Acid-Base Properties of ISFETs and Proteins and the Consequences for the Design of ISFET Biosensors. *Biosens. Bioelectron.* **1995**, *10*, 405–414.
3. Yates, D.E.; Levine, S.; Healy, T.W. Site-Binding Model of Electrical Double-Layer at Oxide-Water Interface. *J. Chem. Soc.-Faraday Trans. I* **1974**, *70*, 1807–1818.
4. Bedner, K.; Guzenko, V.A.; Tarasov, A.; Wipf, M.; Stoop, R.L.; Just, D.; Rigante, S.; Fu, W.; Minamisawa, R.A.; David, C.; *et al*. pH Response of Silicon Nanowire Sensors: Impact of Nanowire Width and Gate Oxide. *Sens. Mater.* **2013**, *25*, 567–576.
5. Knopfmacher, O.; Tarasov, A.; Fu, W.; Wipf, M.; Niesen, B.; Calame, M.; Schoenenberger, C. Nernst Limit in Dual-Gated Si-Nanowire FET Sensors. *Nano Lett.* **2010**, *10*, 2268–2274.
6. Go, J.; Nair, P.R.; Reddy, B.J.; Dorvel, B.; Bashir, R.; Alam, M.A. Beating the Nernst limit of 59mV/pH with double-gated nano-scale field-effect transistors and its applications to ultra-sensitive DNA biosensors. *IEEE Int. Electron Devices Meet.* **2010**, 8.7.1–8.7.4.
7. Ahn, J.-H.; Choi, S.-J.; Han, J.-W.; Park, T.J.; Lee, S.Y.; Choi, Y.-K. Investigation of Size Dependence on Sensitivity for Nanowire FET Biosensors. *IEEE Trans. Nanotechnol.* **2011**, *10*, 1405–1411.
8. Accastelli, E.; Cappi, G.; Buckley, J.; Ernst, T.; Guiducci, C. Comparison between front- and back-gating of Silicon Nanoribbons in real-time sensing experiments. In Proceedings of the IEEE Conference on Nanotechnology, Beijing, China, 5–8 August 2013; pp. 517–520.
9. Vu, X.T.; GhoshMoulick, R.; Eschermann, J.F.; Stockmann, R.; Offenhaeusser, A.; Ingebrandt, S. Fabrication and application of silicon nanowire transistor arrays for biomolecular detection. *Sens. Actuators B Chem.* **2010**, *144*, 354–360.

10. Campbell, J.P.; Cheung, K.P.; Suehle, J.S.; Oates, A. A Simple Series Resistance Extraction Methodology for Advanced CMOS Devices. *IEEE Electron Device Lett.* **2011**, *32*, 1047–1049.
11. Kirchner, J.W. Publisher's note: Aliasing in 1/f(alpha) noise spectra: Origins, consequences, and remedies. *Phys. Rev. E* **2005**, *71*, 066110.
12. MA, C. Microcycle Spectral Estimates of 1-F Noise in Semiconductors. *J. Appl. Phys.* **1974**, *45*, 307–316.
13. Foster, S.; Cranch, G.A.; Tikhomirov, A. Experimental evidence for the thermal origin of 1/f frequency noise in erbium-doped fiber lasers. *Phys. Rev. A* **2009**, *79*, 053802.
14. Kasdin, N.J. Discrete Simulation of Colored Noise and Stochastic-Processes and 1/F(Alpha) Power-Law Noise Generation. *Proc. IEEE* **1995**, *83*, 802–827.
15. Van der Ziel, A. *Noise in Solid State Devices and Circuits*; Wiley-Interscience: Michigan, MI, USA, 1986.
16. Jakobson, C.G.; Nemirovsky, Y. 1/f Noise in Ion Sensitive Field Effect Transistors from Subthreshold to Saturation. *IEEE Trans. Electron Devices* **1999**, *46*, 259–261.
17. Simoen, E.; Veloso, A.; Horiguchi, N.; Claeys, C. Low-Frequency Noise Assessment of the Oxide Quality of Gate-Last High-k pMOSFETs. *IEEE Electron Device Lett.* **2012**, *33*, 1366–1368.
18. Ghibaudo, G.; Roux, O.; Nguyen-Duc, C.; Balestra, F.; Brini, J. Improved Analysis of Low-Frequency Noise in Field-Effect Mos-Transistors. *Phys. Status Solidi A Appl. Res.* **1991**, *124*, 571–581.
19. Simoen, E.; Claeys, C. On the flicker noise in submicron silicon MOSFETs. *Solid-State Electron.* **1999**, *43*, 865–882.
